# Antibiotic resistance of *Mycoplasma Synoviae* strains isolated in China from 2016 to 2019

**DOI:** 10.1186/s12917-021-03104-4

**Published:** 2022-01-03

**Authors:** Xiaorong Zhang, Mengjiao Guo, Di Xie, Yang Chen, Chengcheng Zhang, Yongzhong Cao, Yantao Wu

**Affiliations:** 1 Jiangsu Co-innovation Center for Prevention and Control of Important Animal Infectious Diseases and Zoonoses, College of Veterinary Medicine, Yangzhou University, Yangzhou, 225009 Jiangsu China; 2 Joint International Research Laboratory of Agriculture & Agri-Product Safety, Yangzhou University (JIRLAAPS), Yangzhou, 225009 Jiangsu China

**Keywords:** *Mycoplasma synoviae*, Antimicrobial susceptibility, Minimum inhibitory concentrations, Resistance genes, Enrofloxacin resistance

## Abstract

**Background:**

In the past decade, *Mycoplasma synoviae* (*M. synoviae*) infection has become widely prevalent in China, has caused serious economic losses and has become one of the most important diseases in the chicken industry. Medication is a general approach for the control of *M. synoviae* infection, but antibiotics are sometimes ineffective in clinical practice. To investigate the sensitivity of *M. synoviae* to antimicrobials commonly used in the treatment of *M. synoviae* infection, the antibiotic susceptibility of 32 *M. synoviae* strains isolated from China from 2016 to 2019 were determined using the minimum inhibitory concentration (MIC) method.

**Results:**

All isolates had low MIC values for the combination of lincomycin and spectinomycin, pleuromutilin, and macrolides. However, the *M. synoviae* isolates displayed variance in MICs for doxycycline hydrochloride with a range of 0.25 to 8 μg/mL, and oxytetracycline hydrochloride with a range of 0.5 to 8 μg/mL. Three and one *M. synoviae* isolates showed intermediate MIC values to doxycycline hydrochloride and oxytetracycline hydrochloride, respectively. High MIC values for enrofloxacin were detected in all isolates with MICs ranging from 4 to 32 μg/mL. Furthermore, comparison of the *parC* QRDR identified a mutation at nucleotide position 254 (C254T) resulting in a Thr 85 Ile amino acid change in all *M. synoviae* isolates and the reference strain ATCC 25204 being resistant to enrofloxacin. Moreover, mutations at Glu 804 Gly and Thr 686 Ala of *gyrA* QRDR were identified in all *M. synoviae* isolates and ATCC 25204. The mutation in the QRDR of the *parE* gene resulted in amino acid changes at positions 197 (Pro to Ser) in 27/32 *M. synoviae* isolates.

**Conclusion:**

Three nonsynonymous mutations in *gyrA* and *parE* were first identified to be related to enrofloxacin resistance. Our results showed that *M. synoviae* resistance to enrofloxacin is widespread*.*

## Background

*Mycoplasma synoviae* (*M. synoviae*) is an important pathogen in chickens and turkeys worldwide that can cause acute or chronic infectious synovitis or air sacculitis [1, 2]. In addition, *M. synoviae* infection can also result in a reduction in egg production and eggshell apex abnormalities or subclinical infections in commercial egg layers [3]. Importantly, *M. synoviae* infections can transmit horizontally and vertically. In recent years, *M. synoviae* infection has been widely prevalent in chicken flocks around the world. Previous study in the Netherlands showed that the prevalence of *M. synoviae* was 73.4% (127/173) in commercial layers during 2005 and 2006 [4]. In Portuguese, *M. synoviae* was found in 66.7% (24/36) of broiler breeder flocks between 2008 to and 2012 [5]. Other studies reported a prevalence of 15% in South America [6] and 27% in Middle East [7]. In China, the incidence rate of chickens varies greatly in different provinces from 5.10 to 100%, which has caused serious economic losses to the chicken industry [8]. Therefore, there has been a growing emphasis on the prevalence of *M. synoviae*, especially its subclinical infection.

The three general approaches for the control of *M. synoviae* infection are eradication strategies (maintaining flocks free of infection), vaccination and medication. Although eradication strategies and vaccination provide long-term solutions for the control of *M. synoviae* infection, medication can be very useful in preventing vertical transmission and clinical symptoms as well as economic losses [9]. However, antibiotic susceptibility should first be determined to maximize treatment efficacy. In most countries, excessive use of a range of antimicrobials in feed, on the one hand, prevents and treats disease and, on the other hand, improves growth performance. Antibiotic resistance is a global health threat, and the abuse of antimicrobials in animal production is the main contributing factor [10].

*Mycoplasmas* are readily resistant to β-lactam antibiotics and sulfones, but susceptible to other classes of antibiotics, including tetracyclines, macrolides, fluoroquinolones, and pleuromutilins [11–13]. Previous studies also demonstrated the efficacy of tiamulin fumarate (TIF) and the combination of lincomycin and spectinomycin (LS) against *M. synoviae* [14, 15]. However, increasing resistance of *Mycoplasma* against tetracyclines and quinolones has been reported [16, 17]. Quinolone resistance is related to point mutations in the quinolone resistance-determining regions (QRDRs) of the A subunits of DNA gyrase and topoisomerase IV (*parC* and *gyrA* gene) or B subunits of DNA gyrase and topoisomerase IV (*gyrB* and *parE* gene) in *M. synoviae* [18]*.*

As mentioned above, in recent years, *M. synoviae* infection has caused serious economic losses to the chicken industry. In addition to medication, vaccines also play an important role in controlling *M. synoviae* infection in China. Even when the oil emulsion vaccine is inoculated, it will be mixed with some anti-*Mycoplasma* antimicrobials (such as enrofloxacin and LS) for injection. This has become a common practice in many chicken farms during the brooding period and rearing period. These measures provide great selection pressure for the formation of drug resistance in *M. synoviae*. Our previous study demonstrated that *M. synoviae* strains isolated from China are relatively independent in terms of transmission and evolutionary relationships [19]. The antimicrobial medicines commonly used for the treatment of *M. synoviae* infection are sometimes ineffective in veterinary practice. To screen the most effective antimicrobials, it is urgent to investigate the sensitivity of *M. synoviae.*

## Materials and methods

### Strains

The *M. synoviae* strains used in this study were isolated from China from 2016 to 2019 [19]. The isolates were cultured aerobically at 37 °C in *Mycoplasma* broth (pH = 7.8) with an MB base (OXOID Ltd., Hampshire, UK) containing 15% porcine serum, 3.3 g/L glucose, 100 mg/L L-cysteine, 400 mg/L L-arginine, 100 mg/L β-nicotinamide adenine dinucleotide trihydrate (β-NAD), and 0.02% phenol red until the color of the culture medium changed from red to orange–yellow. These cultures were subsequently titrated in *Mycoplasma* broth medium to determine the number of color changing units (ccu). Then, sterile glycerol (5%) was added to the cultures and stored at − 70 °C.

### Antimicrobials

All strains were tested by the MIC method using the antimicrobials commonly used for *M. synoviae* treatment in farms as follows. LS, doxycycline hydrochloride (DO), valnemulin hydrochloride (VA), tylosin (TY), TIF, oxytetracycline hydrochloride (OT), enrofloxacin (ENR) and tilmicosin (TIL) were purchased from Solarbio Life Sciences (Beijing, China). The concentration of antibiotic solution was diluted to 128 μg/mL. Then the solutions were sterilized with a 0.22-μm membrane filter. The concentration range of antibiotics is shown in Table 3.

### Antimicrobial sensitivity test in vitro

The liquid MIC test was carried out in 96-well microdilution plates as described in a previous study [20]. The *M. synoviae* culture was diluted in *Mycoplasma* broth medium in the range of 10^3^ ccu/mL and 10^5^ ccu/mL. The concentration of antibiotic dilution was usually obtained by doubling dilutions with 100 μL *Mycoplasma* broth medium (pH = 7.8). After the dilution of antibiotic was completed, the 100 μL dilution *M. synoviae* culture was inoculated into each well. *M. synoviae* culture and antibiotics were included in all tests as negative controls and antibiotic controls, respectively. Plates were incubated at 36 ± 1 °C. The lowest concentration of antibiotic to show a color change denoted MIC. The MIC was read when the phenol red indicator in the negative control had just turned orange–yellow. There is a lack of standardized methods and official breakpoints of susceptibility testing criteria for animal mycoplasmas in vitro*.* Thus, MIC values measured in this study were compared to previous studies.

### Quinolone resistance-determining regions and nucleotide sequence analysis

To elucidate the mechanism of acquired ENR resistance in *M. synoviae* isolates, the sequences *gyrA*, *gyrB*, *parC*, and *parE* of QRDRs were further analyzed. To amplify the QRDRs of *M. synoviae* isolates, the primers of *gyrA*, *gyrB*, *parC*, and *parE* (Table 1) were based on a previous study [17]. *M. synoviae* isolate cultures were harvested for DNA extraction using the TIANamp Bacteria DNA Kit (Transgen Biotech Co., Ltd., Beijing, China) according to the manufacturer’s instructions. The sequences were aligned using Lasergene 7.1 software and blasted with those of reference *M. synoviae* strains MS-H (GenBank accession number: NZ_KP704286) and ATCC 25204 (GenBank accession number: NZ_CP011096). The nucleotide and amino acid positions were located based on the reference *M. synoviae* strain MS53 (GenBank accession number: AE017245).

**Table 1:** Primer sequences used in this study

Primer name	Sequences (5′ → 3′)	Position (bp)
*gyrA* F	GAAGATCAGCCTGAATTAGTT	58–78
*gyrA* R	GCCATTCTAGCTTCGGTATAA	531–551
*gyrB* F	CAAGGTGAGAAATTCTCAAGA	964–984
*gyrB* R	TGTGCTTCGTTATAAGCG	1677–1694
*parC* F	CCAACCGTGCAATTCCTGAT	95–114
*parC* R	TTATGCGGCGGCATTTCG	546–563
*parE* F	GGCATATCGTCGAGGAAATAGC	1034–1055
*parE* R	AGTGGTTTCCCAAAGTTG	1741–1758

## Results

### MIC determinations

In this study, 32 *M. synoviae* isolates were tested for antibiotic susceptibility in vitro with 9 commonly used antimicrobials. The MICs of 32 *M. synoviae* isolates was shown in Table 2. All isolates had low MIC values for LS, with MICs ranging from 0.063 to 2 μg/mL. An unimodal distribution of LS MIC value was observed. Thirty-one *M. synoviae* isolates showed MIC values for LS equal or lower to 1 μg/mL (MIC50 = 0.5 μg/mL), while the rest were inhibited by a concentration of 2 μg/mL (Table 3). For pleuromutilin, the *M. synoviae* isolates maintained low MIC values for both VA (MIC < 0.016 ~ 0.031 μg/mL) and TIF (MIC < 0.016 ~ 0.063 μg/mL). Especially, most of the *M. synoviae* (31/32) had MIC values equal or lower than the lowest concentration of VA (0.016 μg/mL), only 1 isolate had a MIC to 0.031 μg/mL. The *M. synoviae* MICs for TY ranged from < 0.016 to 1 μg/mL (MIC50 = 0.125 μg/mL), which was the same as TIL (MIC50 = 0.063 μg/mL), a macrolide antibiotic. However, the *M. synoviae* isolates displayed variance in MICs for tetracyclines, and intermediate MIC results were detected. MIC values for DO and OT, 3/32 and 1/32 *M. synoviae* strains had the highest value at 8 μg/mL. The MIC50 and MIC90 values for DO were same as OT, being 2 and 4 μg/mL respectively (Table 3). Importantly, high MIC values for ENR were detected in all *M. synoviae* isolates with MICs ranging from 4 to 32 μg/mL. ENR MIC values were distributed unimodally and revealed that most of the *M. synoviae* (28/32) were inhibited by concentrations of ≥8 μg/mL, with 12 isolates and 3 isolates showing MIC values of 16 μg/mL and 32 μg/mL respectively. MIC50 and MIC90 values of ENR were the highest, which were 8 μg/mL and 16 μg/mL respectively (Table 3).

**Table 2:** MICs of *M. synoviae* isolates

Isolates	MIC (μg/mL)							
	LS	VA	TIF	TY	TIL	DO	OT	ENR
Ningxia/2019–1	0.5	< 0.016	< 0.016	0.063	< 0.016	4	1	4
Ningxia/2019–2	0.5	< 0.016	< 0.016	0.031	< 0.016	4	2	4
Hebei/2016–1	0.5	< 0.016	0.031	0.25	0.063	4	4	16
Hebei/2016–2	0.5	< 0.016	< 0.016	0.063	0.031	1	2	8
Hebei/2016–3	0.5	< 0.016	< 0.016	0.063	0.031	4	2	8
Shandong/2016–1	1	< 0.016	0.063	0.25	0.063	8	4	16
Shandong/2017–1	0.5	< 0.016	< 0.016	< 0.016	< 0.016	4	4	16
Shandong/2017–2	0.25	< 0.016	0.031	0.063	0.063	2	4	8
Shandong/2018–1	0.5	< 0.016	< 0.016	0.031	0.031	1	1	8
Hubei/2016–1	2	< 0.016	< 0.016	0.063	0.031	2	4	8
Jiangsu/2018–1	0.5	< 0.016	0.031	1	1	1	1	16
Jiangsu/2018–2	0.5	< 0.016	0.031	1	0.5	0.5	0.5	16
Jiangsu/2018–3	1	< 0.016	0.063	0.5	1	1	1	32
Jiangsu/2018–4	0.25	< 0.016	< 0.016	0.25	0.5	1	0.5	8
Jiangsu/2018–5	0.5	< 0.016	< 0.016	< 0.016	0.125	0.5	0.5	4
Jiangsu/2018–6	1	< 0.016	0.031	0.5	1	1	0.5	16
Anhui/2019–1	1	< 0.016	0.031	0.125	0.031	4	4	16
Anhui/2019–2	1	< 0.016	0.063	0.5	0.125	1	1	32
Jiangsu/2019–1	0.063	< 0.016	< 0.016	< 0.016	< 0.016	1	2	8
Shandong/2019–2	0.5	< 0.016	< 0.016	0.063	0.063	0.25	0.5	16
Shandong/2019–3	1	< 0.016	0.063	0.125	0.5	1	2	32
Shandong/2019–4	0.125	< 0.016	< 0.016	< 0.016	< 0.016	2	4	8
Ningxia/2019–3	0.125	< 0.016	< 0.016	0.125	< 0.016	2	2	8
Henan/2019–1	1	< 0.016	0.063	0.063	0.125	2	2	16
Jiangsu/2019–2	0.25	< 0.016	< 0.016	0.25	0.5	0.5	0.5	8
Jiangsu/2019–3	0.5	< 0.016	0.031	0.5	0.5	1	0.5	16
Ningxia/2019–4	0.5	< 0.016	0.031	0.25	0.063	8	2	8
Ningxia/2019–5	0.5	< 0.016	0.031	0.25	0.063	4	4	16
Heilongjiang/2019–1	0.5	0.031	0.031	0.125	0.063	4	2	8
Hebei/2018–1	0.125	< 0.016	< 0.016	0.031	0.031	2	2	8
Hebei/2018–2	1	< 0.016	0.063	0.125	0.031	8	8	16
Shandong/2019–5	0.125	< 0.016	< 0.016	0.063	< 0.016	2	1	4

**Table 3:** Distribution of MIC values of the tested antimicrobials against the 32 *M. synoviae* isolates

Antimicrobials	MIC Values (μg/mL)												
	0.016	0.031	0.063	0.125	0.25	0.5	1	2	4	8	16	32	64
LS			1	4	3	15^50^	8^90^	1					
VA	31^50–90^	1											
TIF	16^50^	10	6^90^										
TY	4	3	8	5^50^	6	4^90^	2						
TIL	7	7	7^50^	3		5^90^	3						
DO					1	3	10	7^50^	8^90^	3			
OT						7	6	10^50^	8^90^	1			
ENR									4	13^50^	12^90^	3	

The MIC values are expressed in μg/mL. Superscript numbers indicate the MIC50 and MIC90 values

### Molecular characterization of quinolone resistance-determining regions in *M. synoviae* isolates

To further investigate the mechanism of ENR resistance in *M. synoviae*, four resistance genes in the QRDRs of 32 *M. synoviae* isolates were amplified by PCR and sequenced. The reference strain ATCC 25204 is resistant to ENR and MS-H is susceptible to ENR. Four QRDR genes in the reference genome of *M. synoviae* strain MS53 were aligned with the sequences of ATCC 25204, MS-H and 32 *M. synoviae* isolates. Several nonsynonymous mutations were found to be potentially resistance-related.

Sequence analysis of the *gyrA* QRDR showed that three SNPs in all *M. synoviae* isolates and ATCC 25204 resistant to ENR were identified. Mutations at nucleotide positions 2410 (A2410G) and 2411 (A2411G) resulted in Glu 804 Gly amino acid changes. A mutation at nucleotide position 2058 (A2058G) resulted in a Thr 686 Ala amino acid change. In the *gyrB* gene, a SNP mutation was found at position 1250 (G1250A) and resulted in a Ser 417 Asn amino acid change in 11/32 *M. synoviae* isolates with high MIC values for ENR. Comparison of the *parC* QRDR identified a mutation at nucleotide position 254 (C254T) resulting in a Thr 85 Ile amino acid change in all *M. synoviae* isolates and ATCC 25204. A mutation at nucleotide position 591 (C591T) of the *parE* gene resulting in a Pro 197 Ser amino acid change was also found in 27/32 *M. synoviae* isolates (Table 4).

**Table 4:** Molecular characterization of quinolone resistance-determining regions of *M. synoviae* isolates

Strains	*gyrA*						*gyrB*		*parC*		*parE*	
	SNP	AA	SNP	AA	SNP	AA	SNP	AA	SNP	AA	SNP	AA
	2442	814	2410–2411	804	2058	686	1250	417	254	85	591	197
MS-H	C	Gln	AA	Glu	A	Thr	G	Ser	C	Thr	C	Pro
ATCC25204	C	Gln	GG	Gly	G	Ala	G	Ser	T	Ile	T	Ser
Hebei/2016–1	C	Gln	GG	Gly	G	Ala	G	Ser	T	Ile	T	Ser
Hebei/2016–2	C	Gln	GG	Gly	G	Ala	G	Ser	T	Ile	T	Ser
Hebei/2016–3	C	Gln	GG	Gly	G	Ala	G	Ser	T	Ile	T	Ser
Hebei/2018–1	C	Gln	GG	Gly	G	Ala	G	Ser	T	Ile	T	Ser
Hebei/2018–2	C	Gln	GG	Gly	G	Ala	G	Ser	T	Ile	T	Ser
Jiangsu/2018–1	C	Gln	GG	Gly	G	Ala	A	Asn	T	Ile	T	Ser
Jiangsu/2018–2	C	Gln	GG	Gly	G	Ala	A	Asn	T	Ile	T	Ser
Jiangsu/2018–3	C	Gln	GG	Gly	G	Ala	A	Asn	T	Ile	T	Ser
Jiangsu/2018–4	C	Gln	GG	Gly	G	Ala	A	Asn	T	Ile	T	Ser
Jiangsu/2018–5	C	Gln	GG	Gly	G	Ala	A	Asn	T	Ile	T	Ser
Jiangsu/2018–6	C	Gln	GG	Gly	G	Ala	A	Asn	T	Ile	T	Ser
Jiangsu/2019–1	C	Gln	GG	Gly	G	Ala	G	Ser	T	Ile	T	Ser
Jiangsu/2019–2	C	Gln	GG	Gly	G	Ala	A	Asn	T	Ile	T	Ser
Jiangsu/2019–3	C	Gln	GG	Gly	G	Ala	A	Asn	T	Ile	T	Ser
Ningxia/2019–1	C	Gln	GG	Gly	G	Ala	G	Ser	T	Ile	T	Ser
Ningxia/2019–2	C	Gln	GG	Gly	G	Ala	G	Ser	T	Ile	T	Ser
Ningxia/2019–3	C	Gln	GG	Gly	G	Ala	G	Ser	T	Ile	T	Ser
Ningxia/2019–4	C	Gln	GG	Gly	G	Ala	G	Ser	T	Ile	T	Ser
Ningxia/2019–5	C	Gln	GG	Gly	G	Ala	G	Ser	T	Ile	T	Ser
Shandong/2016–1	C	Gln	GG	Gly	G	Ala	G	Ser	T	Ile	T	Ser
Shandong/2017–1	C	Gln	GG	Gly	G	Ala	G	Ser	T	Ile	T	Ser
Shandong/2017–2	C	Gln	GG	Gly	G	Ala	G	Ser	T	Ile	C	Pro
Shandong/2018–1	A	Lys	GG	Gly	G	Ala	G	Ser	T	Ile	T	Ser
Shandong/2019–2	C	Gln	GG	Gly	G	Ala	A	Asn	T	Ile	T	Ser
Shandong/2019–3	C	Gln	GG	Gly	G	Ala	G	Ser	T	Ile	C	Pro
Shandong/2019–4	C	Gln	GG	Gly	G	Ala	G	Ser	T	Ile	T	Ser
Shandong/2019–5	C	Gln	GG	Gly	G	Ala	A	Asn	T	Ile	T	Ser
Anhui/2019–1	C	Gln	GG	Gly	G	Ala	G	Ser	T	Ile	C	Pro
Anhui/2019–2	C	Gln	GG	Gly	G	Ala	A	Asn	T	Ile	T	Ser
Henan/2019–1	C	Gln	GG	Gly	G	Ala	G	Ser	T	Ile	C	Pro
Hubei/2016–1	C	Gln	GG	Gly	G	Ala	G	Ser	T	Ile	C	Pro
Heilongjiang/2019–1	C	Gln	GG	Gly	G	Ala	G	Ser	T	Ile	T	Ser

## Discussion

Antimicrobial resistance is a growing global concern for animals and humans. During the past decade, few studies have investigated the MICs of *M. synoviae* isolates in vitro. At present, the commonly used clinical antimicrobial medicines for the treatment of mycoplasma disease are macrolides (e.g., TY and TIL), pleuromutilins (e.g., valnemulin and TIF), tetracyclines (e.g., DO and OT), fluoroquinolones (e.g., ENR), as well as LS. In this study, we investigated the antimicrobial susceptibility of 32 *M. synoviae* strains isolated from China from 2016 to 2019.

Our results showed that the MIC values of *M. synoviae* isolates were generally low for LS, pleuromutilin, and macrolides. Similar results were observed in a recent study in Asia, except for TIL [21]. When spectinomycin was applied in combination with lincomycin, it improved the efficacy of two antimicrobials against most *M. synoviae* [22]. In this study, 31/32 isolates were sensitive to low concentrations of LS (MICs 0.063 ~ 1 μg/mL). A previous study investigated the antibiotic susceptibility of 41 *M. synoviae* strains originating from Central and Eastern Europe between 2002 and 2016, including Hungary, Austria, the Czech Republic, Slovenia, Ukraine, Russia, and Serbia. Overall, similar low MIC values (0.25 ~ 2 μg/mL) were detected for LS [13]. The macrolides showed good activity against *M. synoviae* strains worldwide, but higher MIC values (> 2 μg/mL) were also identified in Europe [13, 23, 24]. In the current study, all isolates were sensitive to low concentrations of TY and TIL with MICs of only 2/32 and 3/32 isolates = 1 μg/mL. In contrast, TIL MICs clearly showed a time-dependent gradual transition to high concentrations in 154 *M. synoviae* isolates from Italy collected from 2012 to 2017. Seven *M. synoviae* isolates showed an MIC > 32 μg/mL for TIL between 2013 and 2016 [25]. High MIC values were also detected in another study, which showed 25/87 *M. synoviae* strains with high MIC values (> 8 μg/ml for TIL and/or > 1 μg/ml for TY and/or > 0.5 μg/ml for tylvalosin) from 18 different countries from 1982 to 2019 [26]. As mentioned above, increased TIL MICs (≥64 μg/ml MIC_90_ values) were also detected in *M. synoviae* isolates collected from China, India, Indonesia, Malaysia, the Philippines, the Republic of Korea, and Thailand [21]. Our results confirmed the high efficiency of TY and TIL against *M. synoviae* in China. Previous research has shown that pleuromutilins display high efficacy against avian *mycoplasmas* [27]. To date, the MIC values for TIF in the Europe mentioned above are relatively low (0.004 ~ 2.5 μg/mL). All *M. synoviae* isolates remained sensitive to TIF with MICs ranging from 0.12 to 2.5 μg/mL in South Africa between 2003 and 2015 [16]. The MIC values of VA were ≤ 0.039 μg/mL in Central and Eastern Europe [26]. The *M. synoviae* isolates examined in this study showed high susceptibility to VA (MIC < 0.016 ~ 0.031 μg/mL) and TIF (MIC < 0.016 ~ 0.063 μg/mL). Especially, 31 *M. synoviae* isolates had MIC values equal or lower than the lowest concentration of VA (0.016 μg/mL). Therefore, pleuromutilins are supposed to be preferable in the treatment of *M. synoviae* infection.

For tetracyclines, 3/32 of *M. synoviae* isolates showed intermediate MIC values for DO, and only one strain showed an intermediate MIC value for OT. The MIC50 and MIC90 values for DO were same as OT, being 2 and 4 μg/mL respectively. The finding of tetracycline resistance was not unexpected because of the long-term widespread use of tetracyclines in feed in China. Aureomycin, DO and OT are the most widely used antimicrobials. It can not only prevent bacterial infection but also improve the growth performance of animals. Our results indicated that long-term use of tetracycline antimicrobials can reduce the sensitivity of *M. synoviae*. Interestingly, even though the *M. synoviae* isolates did not have high MIC values of tetracyclines, our results do not align with previous studies in Europe, which showed that the MIC values of OT were higher than those of DO. For example, MIC values of OT and DO to *M. synoviae* were 0.031 ~ 32 μg/mL and 0.062 ~ 2 μg/mL, respectively, from six European countries from 2014 to 2016 [24]. In another study, 84 *M. synoviae* field strains were collected from 18 different countries from 2010 to 2019, and the majority of strains were from Hungary, Italy, the Netherlands, Israel, and Spain. The MIC values of OT and DO were ≤ 0.25 ~ 8 μg/mL and ≤ 0.039 ~ 1.25 μg/mL, respectively [26]. The difference in MIC value may be due to the geographic area, density of poultry flocks, and different quantitative uses among countries. Since 2020, all forms of growth-promoting antimicrobials, except for traditional Chinese medicines have been forbidden to be used as feed additives in China. With the increase in the number of laws and regulations concerning the use of antimicrobials, we speculate that the resistance of *M. synoviae* to tetracyclines may be decreased in the future.

Previous studies showed that resistance to ENR increased rapidly [17, 28, 29]. In this study, high MIC values (4 ~ 32 μg/mL) for ENR were present in all *M. synoviae* isolates. The MIC50 and MIC90 values of ENR were the highest, with 12 isolates showing MIC values of 16 μg/mL, 3 isolates showing MIC values of 32 μg/mL. In Israel and Europe, decreased susceptibility to ENR was detected in 59% of *M. synoviae* field strains, with MICs ranging from 1 to > 16 μg/mL [17]. There is no standardized method for MIC testing in animal *mycoplasma*, but genetic mutations can determine the presence of antimicrobial resistance. To further investigate the mechanism of ENR resistance, *gyrA*, *gyrB*, *parC*, and *parE* genes in the QRDRs of 32 MS isolates were sequenced and analyzed. Topoisomerase IV (*parC*) is considered to be the primary target of ENR in *M. synoviae*, based on decreased susceptibility after experimental infection in vivo [18]. Amino acid positions 85–89 (80–84 according to *Escherichia coli* strain K-12 substrain MG1655) of *parC* were identified as hot spot regions that seem to have a principal role [26]. Amino acid substitutions at positions 80 and 84 of *parC* are known as important spots for ENR resistance in many bacteria, including mycoplasmas and may be alone or together with a mutation of *gyrA* [30–33]*.* In addition, amino acid substitutions at positions 79 and 81 of *parC* were also identified in mycoplasma [17, 34]. In the current study, comparison of the *parC* QRDR identified a mutation at nucleotide position 254 (C254T) resulting in a Thr 85 Ile amino acid change in all *M. synoviae* isolates and ATCC 25204, which was similar to a previous study [26]. In the *gyrB* gene, a SNP mutation has been found at position 1250 (G1250A) and resulted in a Ser 417 Asn amino acid change in 11/32 *M. synoviae* isolates with high MIC values to ENR, which has also been reported [17].

Comparison of the *gyrA* QRDR found the presence of different amino acid substitutions at positions 686, 804 and 814. Mutations at Glu 804 Gly and Thr 686 Ala were identified in all *M. synoviae* isolates and ATCC 25204. To our knowledge, these nonsynonymous mutations have not been reported in previous research. Our results revealed that there is a correlation between MIC values and amino acid mutations at positions 804 and 686 of the *gyrA* QRDR. Indeed, *M. synoviae* isolates containing these two amino acid substitutions had MICs ranging from 4 to 32 μg/mL. These two amino acid mutations may together affect *M. synoviae* with decreased susceptibility to ENR. More strains with a broader spectrum of MICs should be identified to prove this conclusion, and the relevance of the mutations that occurred in *gyrA* QRDR should be further investigated. The mutation in the QRDR of *parE* at positions 197 (Pro to Ser) in 27/32 *M. synoviae* isolates has also not been reported. Amino acid substitution at position 420 of *parE* corresponds to residue 426 Asp of *gyrB* in *E. coli*, which is a multiple possible marker for quinolone resistance in many bacteria [35, 36]. The role of position 197 of *parE* in quinolone resistance needs to be further established.

In conclusion, 32 *M. synoviae* isolates had low MIC values for the combination of lincomycin and spectinomycin, pleuromutilin and macrolides. However, 3/32 and 1/32 *M. synoviae* isolates showed intermediate MIC values for DO and OT. High MIC values for ENR were detected in all isolates, with MICs ranging from 4 to 32 μg/mL. Furthermore, mutations at Glu 804 Gly and Thr 686 Ala of *gyrA* QRDR were identified in all *M. synoviae* isolates and ATCC 25204. The mutation in the QRDR of the *parE* gene resulted in amino acid changes at positions 197 (Pro to Ser) in 27/32 *M. synoviae* isolates. These nonsynonymous mutations were first identified to be related to ENR resistance.

## Data Availability

The datasets used and/or analysed during the current study available from the first author (E-mail: zxr@yzu.edu.cn) on reasonable request.
